# Ranolazine Versus Allopurinol for Eligible Symptomatic Patients With a History of Angioplasty: Comparative Efficacy Study

**DOI:** 10.2196/39778

**Published:** 2022-08-17

**Authors:** Reza Rahmani, Ehsan Moradi Farsani, Sima Bahrami

**Affiliations:** 1 Tehran University of Medical Sciences Tehran Iran

**Keywords:** ranolazine, allopurinol, recurrent angina, exercise tolerance

## Abstract

**Background:**

Recurrent angina, which is defined as a return of chest pain or chest discomfort, occurs in many patients undergoing coronary interventions.

**Objective:**

This study aims to compare the antianginal efficacy of ranolazine versus allopurinol for eligible symptomatic patients with a history of angioplasty.

**Methods:**

A total of 62 eligible symptomatic patients with a history of angioplasty were randomly allocated into two groups. For group A, 300 mg of allopurinol was administered twice daily, while for group B, 1000 mg of ranolazine daily was prescribed for a duration of 4 weeks. An initial screening visit was done for all participants where patients’ medical history was recorded and a physical examination was given; electrocardiography, blood pressure, and heart rate measurements were done as well. The patients were also given a blood and exercise test. At the end of the medication period, participants were revisited, and the tests were done again. All the required data were collected via a researcher-made form, and data analysis was conducted using SPSS. The study was approved by a formal ethics committee.

**Results:**

The mean age of participants in the two groups (A and B) was 57.36 (SD 8.36) and 60.27 (SD 9.17) years, respectively. Among the 62 patients, 34 (59%) were men, while 28 (41%) were women. Creatinine, fasting blood sugar, C-reactive protein, N-terminal prohormone of brain natriuretic protein, uric acid, white blood cell, and hemoglobin levels of participants were not significantly different between groups (*P*>.05). Both allopurinol and ranolazine increased the total exercise time and decreased the ST depression of the patients. Additionally, they both improved the chest pain severity and Duke Treadmill Score of patients. At the same time, ranolazine had a statistically greater effect on ST depression reduction (mean 2.64, SD 0.74 vs mean 1.57, SD 0.49), while allopurinol showed better efficacy in reducing chest pain severity (mean 1.86, SD 0.37 vs mean 0.59, SD 0.21) and the Duke Treadmill Score (mean –14.77, SD 3.65 vs mean –6.88, SD 1.93).

**Conclusions:**

Based on the results, the antianginal efficacy of allopurinol and ranolazine was approved but with different effects on ST depression, chest pain severity, and the Duke Treadmill Score. Therefore, the precise differences in their effects need to be explored further.

## Introduction

Cardiovascular diseases are considered as the most common cause of death and disability in most countries. For the health systems, prevention and treatment of diseases are among the main priorities [1]. Treatments of coronary artery diseases include medication and intravascular interventions in addition to controlling risk factors. Intravascular interventions include coronary artery bypass graft surgery and percutaneous vasodilation [2]. Coronary angioplasty, as one of the most influential advances in the treatment of patients with coronary heart disease, can substantially reduce the need for vascular transplant surgery. However, restenosis of the coronary arteries after angioplasty is one of the most important concerns of cardiologists. In various studies, the rate of restenosis of arteries 6 months after angioplasty has been reported in about half of the cases [3]. To solve this problem, interventional cardiologists have introduced intracoronary stents, which is also an important breakthrough in the treatment of heart diseases. Numerous studies have shown that the rate of restenosis after stent implantation is significantly lower than balloon angioplasty in the first 6 months after the intervention [3-6]. Unfortunately, the introduction of this method has not completely solved the problem of coronary artery restenosis after the intervention. Existing studies show that restenosis and experiencing recurrent angina occur in a significant proportion of patients receiving cardiovascular stents [2,7-10]. Numerous factors such as older age, more stents, diabetes, and the use of aspirin and beta-blockers increase the chances of recurrence [11].

Recurrent angina is defined as the recurrence of chest pain or chest discomfort in patients undergoing coronary intervention. Due to the possible complications of artery restenosis after stenting, timely diagnosis and treatment plays an important role in patients’ survival and quality of life (QOL) [12]. Although so far no specific diagnostic algorithm has been developed for these cases, electrocardiography, an exercise test, a stress-level detection test, or invasive coronary assessment may be required. However, some diagnostic methods such as invasive coronary angiography or noninvasive scanning are not routinely available due to the potential complications of invasive interventions as well as the high cost. Therefore, specialists often use exercise tests and clinical signs to diagnose restenosis [3,13]. To treat this complication, various options such as balloon angioplasty, restenting, rotational atherectomy, directional atherectomy, excimer laser angioplasty, intracoronary radiation therapy, or pharmacological methods are suggested. In cases where vascularization is not required, treatment approaches with a variety of available medications are used. Today, various medications including ranolazine and allopurinol are available for this purpose, and their efficacy has also been studied in numerous papers [3,6,10]. For instance, Chaitman et al [10] examined the effect of ranolazine in combination with amlodipine or diltiazem on exercise tolerance and angina recurrence in patients with chronic angina. Noman et al [9] investigated the effect of high-dose allopurinol on exercise tolerance in patients with chronic angina using a randomized controlled trial with a placebo. Chaturvedi et al [14] in a follow-up open labeled trial examined the efficacy and tolerability of ivabradine and ranolazine in patients with chronic stable angina pectoris. Al-Zahrani et al [15] studied the possible antianginal effect of allopurinol in a vasopressin-induced ischemic model in rats through an experimental study. Rousseau et al [16] have also compared the efficacy of ranolazine versus atenolol for chronic angina pectoris.

Despite this, and even though drug treatments in some studies have been compared, it is still not clear which medication regimen has the most favorable result. Therefore, suggesting appropriate drug treatment for patients with stable angina after stenting intervention requires further studies [6]. This study aimed to compare the efficacy of ranolazine versus allopurinol for eligible symptomatic patients with a history of angioplasty. To the best of our knowledge, there are some studies on the efficacy of ranolazine or allopurinol separately. Additionally, other studies have compared the efficacy of one of these with another drug, but the efficacy of ranolazine has not been compared with allopurinol in any previous study. Indeed, allopurinol has been reported as an inexpensive, well-tolerated, and safe option that should be studied further. As Noman et al [9] mention, the precise place of allopurinol in the management of angina pectoris needs to be explored further, but this drug might be especially appealing for use in low-income countries where coronary artery disease is rapidly increasing and where access to expensive drugs or invasive treatments is often restricted [9]. Therefore, comparison of these drugs’ efficacy provides new insights for the clinicians. This was the main novelty of this study.

## Methods

### Setting

A clinical trial was conducted at Imam Khomeini Hospital, Tehran, Iran.

### Participants

The study population included patients with a history of stenting but who still had the coronary clinical symptoms after the intervention and needed medical treatment.

### Inclusion and Exclusion Criteria

Inclusion criteria are defined as follows: having a positive exercise tolerance test or limited capacity in the treadmill test based on the modified Bruce protocol (patients who had a total exercise time between 3 and 9 minutes); at least 6 months have passed since stenting; and having a history of chronic, recurrent, and stable angina after stent implantation. The exclusion criteria considered factors that prevented the correct interpretation of the electrocardiogram, grade 3 or 4 heart failure, intravascular reintervention in the last 6 months, the inability to perform exercise test due to leg and back problems, a history of acute myocardial infarction, a history of acute vascular syndrome, left ventricular rejection fraction less than 45, estimated glomerular rate less than 45 mm per minute, creatinine concentration greater than 180 mmol/ml, significant heart valve disease, atrial arrhythmia, electrocardiogram abnormalities that interfere with ST segment interpretation, and using the study drugs (allopurinol or ranolazine). Additionally, patients who had a total exercise time of less than 3 minutes, those who did not want to continue participation, and those who needed invasive cardiovascular intervention during the study were excluded.

### Randomization and Blinding

All the participants were randomly divided into two groups: allopurinol and ranolazine. Randomization was done by random number generation in 12 blocks using SPSS Version 22 (IBM Corp) software.

For blinding, all visits, exercise tests, and clinical investigations of participants were performed under the supervision of a research team member who was unaware of the treatment allocation. The preparation of numbered medicine containers (allopurinol or ranolazine) was also done by a member of the research team who was not aware of the drug allocation and the results of the patients’ tests. Random numbers and drug assignments were also created by a staff member of the hospital who was not a member of the research team. Additionally, other members of the research team and patients had no access to the assignment sequence.

### Sample Size

For the sample size calculation, we anticipated a detectable 30-second difference of total exercise test tolerance time between the 2 groups based on the pilot analysis of 12 patients and the reports of previous studies [9,10]. The required sample size was then calculated as 58 (29 samples for each group) using a test power of 90% and an allocation ratio of 1. Finally, 62 patients were included for further analysis.

### Procedure

A total of 62 patients participated in the study. First, the objective of the study was explained for the patients and an informed written consent was obtained. An initial screening visit was done for all the participants. In this visit, patients’ medical history was recorded; a physical examination was carried out; and electrocardiography, blood pressure, and heart rate measurements were done. Additionally, a blood test including creatinine, fasting blood sugar, C-reactive protein, N-terminal prohormone of brain natriuretic protein, uric acid, white blood cell, and hemoglobin was carried out on the patients. In addition, an exercise test was performed by all patients. Patients who had a positive exercise test or a limited capacity treadmill test based on the modified Bruce protocol were eligible to participate in the study [17]. We included those patients who had the total exercise time between 3 to 9 minutes. For the allopurinol group, 300 mg twice daily was administered, and for the ranolazine group, 1000 mg daily was administered for 4 weeks. During the study, patients were allowed to take their underlying medications without modification. At the end of the medication period, participants were revisited. In this visit, patients’ medical history was recorded; a physical examination was carried out; and electrocardiography, blood pressure, and heart rate measurements were done. Additionally, a blood test was carried out for the participants. In addition, for all patients, an exercise test was performed at peak times (4 hours after medication). Drug reactions were followed for all the patients, but no adverse reaction was documented.

### Analysis

All the required data was collected via a researcher-made form. It should be noted that conducting the research did not incur any additional financial costs for patients. The collected data was analyzed using SPSS statistical software. Descriptive statistics (frequency, percentage, mean, and SD) and independent sample *t* test were used for analysis.

### Ethics Approval

All participants provided an informed written consent to participate in the study and were assured that their information would be kept confidential. All study procedures were conducted in accordance with the ethical standards of the Declaration of Helsinki. The study was approved by the ethics committee affiliated with the Tehran University of Medical Sciences (IR.TUMS.IKHC.REC.1399.315).

## Results

**Figure 1:**
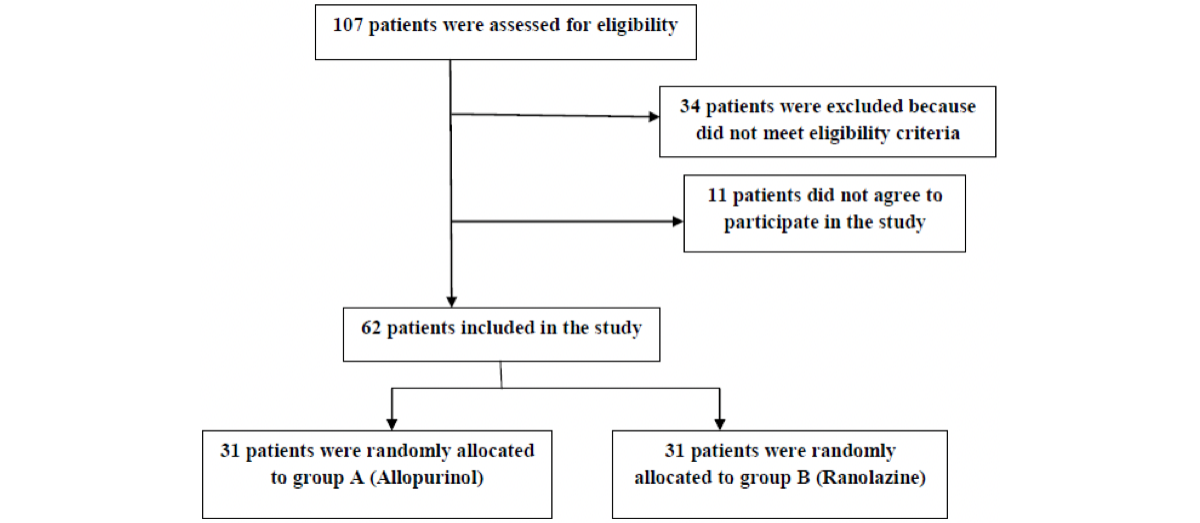
The study procedure.

A total of 62 patients in 2 groups, allopurinol (group A) and ranolazine (group B), participated in the study. Figure 1 shows the study procedure.

As Figure 1 illustrates, a total of 107 patients were assessed for eligibility, from which 62 patients were allocated to the study groups randomly. The baseline characteristics of participants are presented in Table 1. All the participants were using aspirin, statin, P2Y12 inhibitor, beta-blocker, nitrates, and calcium blocker, which were continued during the course of the study.

As Table 1 indicates, most of the participants had hypertension or hypercholesterolemia. Additionally, the majority had one stent. The blood test results of participants are presented in Table 2.

As shown in Table 2, the blood test parameters of the 2 groups had no statistical differences. Tables 3 and 4 show the results of the exercise test for the 2 groups and the comparison of the outcomes between them.

Table 3 shows that allopurinol and ranolazine both significantly improved the total exercise time of patients, ST depression, chest pain severity, and Duke Treadmill Score from baseline. Therefore, the efficacy of studied drugs as an antianginal drug was approved.

Table 4 reports the comparison analysis of the studied drugs’ effects on the outcomes. As seen, allopurinol and ranolazine both increased the total exercise time with no statistical difference. However, ranolazine showed better efficacy in improving ST depression and the Duke Treadmill Score, while allopurinol showed higher efficacy in decreasing chest pain severity.

**Table 1:** Baseline characteristics of participants.

			Allopurinol (n=31)	Ranolazine (n=31)
Age (years), mean (SD)			57.36 (8.36)	60.27 (9.17)
**Gender, n (%)**				
	Male		17 (55)	20 (65)
	Female		14 (45)	11 (35)
**Medical history, n (%)**				
	Hypertension		20 (65)	18 (58)
	Diabetes mellitus		11 (35)	8 (26)
	Hypercholesterolemia		19 (61)	23 (74)
	**Number of vessels with coronary artery disease**			
		1	8 (26)	11 (35)
		2	8 (26)	6 (19)
		3	0 (0)	0 (0)
		Multiple	15 (49)	14 (45)
	**Number of stents**			
		1	14 (45)	18 (58)
		2	11 (35)	8 (26)
		3	6 (19)	5 (16)
		>3	0 (0)	0 (0)
**Smoking, n (%)**				
	Yes		6 (19)	9 (29)
	No		27 (82)	22 (71)

**Table 2:** The blood test results of two groups.

Test	Allopurinol, mean (SD)	Ranolazine, mean (SD)	*P* value
Creatinine (*μ*mol/L)	0.89 (0.23)	1.01 (0.17)	.16
FBS^a^ (mg/dl)	103.00 (34.25)	87.36 (18.50)	.20
CRP^b^ (mg/l)	1.54 (0.93)	1.18 (0.60)	.29
NT-pro BNP^c^ (pg/ml)	98.10 (23.76)	102.30 (33.35)	.75
Uric acid (mg/dl)	2.99 (0.68)	3.30 (0.84)	.36
WBC^d^ (×10^9^ per L)	6.16 (1.35)	5.58 (1.12)	.29
HB^e^ (g/dl)	12.66 (1.09)	13.02 (1.00)	.43

^a^FBS: fasting blood sugar.

^b^CRP: C-reactive protein.

^c^Nt-pro BNP: N-terminal prohormone of brain natriuretic protein.

^d^WBC: white blood cell.

^e^HB: hemoglobin.

**Table 3:** The results of the exercise test for the 2 groups.

Variable	Allopurinol, mean (SD)		*P* value	Ranolazine, mean (SD)		*P* value
	Baseline	After		Baseline	After	
Total exercise time (s)	263 (74)	342 (89)	*.03* ^a^	371 (97)	454 (102)	*.02*
ST depression	2.43 (0.92)	1.72 (0.87)	*.04*	2.64 (0.74)	1.57 (0.49)	*.02*
Chest pain severity	1.86 (0.37)	0.59 (0.21)	.*03*	2.11 (0.43)	1.70 (0.46)	*.01*
Duke Treadmill Score	–14.77 (3.65)	–6.88 (1.93)	*.02*	–16.87 (3.89)	–6.72 (2.26)	*.01*

^a^Italics indicate significance at the *P*<.05 level.

**Table 4:** Comparison of the changes of studied outcomes between 2 groups.

Variable	Allopurinol^a^, mean (SD)	Ranolazine^a^, mean (SD)	*P* value
Total exercise time (s)	79 (37)	83 (39)	.18
ST depression	0.71 (0.25)	1.07 (0.28)	*.03* ^b^
Chest pain severity	1.27 (0.29)	0.41 (0.14)	*.02*
Duke Treadmill Score	7.89 (1.76)	10.15 (2.94)	*.01*

^a^Changes of the outcomes after the course of treatment.

^b^Italics indicate significance at the *P*<.05 level.

## Discussion

### Principal Findings

This study aimed to compare the efficacy of ranolazine and allopurinol for eligible symptomatic patients with a history of angioplasty. Based on the findings, ranolazine and allopurinol showed appropriate antianginal efficacy. Both the medications increased total exercise time with no statistical difference. However, ranolazine affected ST depression and the Duke Treadmill Score more than allopurinol, while allopurinol showed a better efficacy in decreasing chest pain severity. Therefore, this study approved the efficacy of both drugs as an antianginal drug like the previous studies. It is recommended to study the different effects on the outcome measures in future studies.

### Comparison to Prior Works

Our study approved the antianginal efficacy of both studied drugs but with some differences. Other studies have confirmed the efficiency of these treatments, but the available studies have reported different results in this area.

Noman et al [9] in a randomized placebo-controlled trial investigated the effect of high-dose allopurinol on exercise in patients with chronic stable angina. The results of this study showed that allopurinol is a useful, inexpensive, well-tolerated, and safe anti-ischemic drug for patients with angina. In this study, administration of allopurinol 600 mg per day was reported to be associated with a 43-second median increase in exercise time to ST-segment depression, a 58-second median increase in total exercise time, and a 38-second median increase in time to chest pain.

Alexander et al [13] in a clinical trial investigated the effects of ranolazine on angina and QOL after percutaneous coronary intervention (PCI) with incomplete revascularization. This study concludes that adding ranolazine in the angiographically identified population has no incremental benefit in angina or QOL measures. Stone et al [18] in the Efficacy of Ranolazine in Chronic Angina (ERICA) trial investigated the antianginal efficacy of ranolazine when added to the treatment with amlodipine in stable coronary patients. They have concluded that ranolazine significantly reduces the frequency of angina compared with the placebo while being well tolerated. Another study, the Type 2 Diabetes Evaluation of Ranolazine in Subjects with Chronic Stable Angina (TERISA) trials have reported a similar result for ranolazine administration in patients with type 2 diabetes mellitus, coronary artery disease, and chronic stable angina who remain symptomatic despite treatment with up to 2 antianginal agents [19]. Wilson et al [20] in a randomized, double-blind, placebo-controlled Metabolic Efficiency With Ranolazine for Less Ischemia in Non–ST-Segment Elevation Acute Coronary Syndromes (MERLIN-TIMI) 36 Trial have evaluated the efficacy and safety of ranolazine in a larger and diverse group of patients, and reported that ranolazine is effective in reducing angina and has favorable safety for patients with angina. Gutierrez et al [21] in another study from the MERLIN-TIMI 36 Trial have studied the effects of ranolazine in patients with chronic angina and showed that, in patients with chronic angina, ranolazine reduces recurrent ischemic events, regardless of whether patients did or did not receive PCI within 30 days of a non–ST-segment for acute coronary syndromes. Sendón et al [22], also in a Combination Assessment of Ranolazine in Stable Angina (CARISA) randomized trial of 258 patients, reported that ranolazine is effective for the symptomatic treatment of patients with stable angina on background therapy with maximally tolerated doses of first-line antianginal therapies.

Chaitman et al [10], in a randomized controlled trial with a placebo, investigated the effects of ranolazine with atenolol, amlodipine, or diltiazem on exercise tolerance and angina frequency in patients with severe chronic angina. The results showed that twice-daily doses of ranolazine increases exercise capacity and provides additional antianginal relief to symptomatic patients with severe chronic angina who are taking standard doses of atenolol, amlodipine, or diltiazem over 1 year to 2 years of therapy. Chaitman et al [5] in a double-blind randomized clinical trial evaluated the anti-ischemic effects and long-term survival during ranolazine monotherapy in patients with chronic severe angina. They concluded that ranolazine is well-tolerated and improves exercise performance without any significant adverse effect. Timmis et al [23] studied the effects of ranolazine on exercise tolerance and hemoglobin A_1c_ in patients with chronic angina and diabetes. They concluded that ranolazine makes similar improvements in exercise parameters, nitroglycerin use, and angina frequency in patients who are diabetic and nondiabetic.

### Strengths and Limitations

Our study had some limitations alongside its strengths and applications. First, our sample size was small; although we used the results of previous studies to calculate the needed sample size, the study could be conducted with larger samples. The main cause of this limitation was the sampling restrictions caused by the COVID-19 pandemic. Second, screening of HLA B*5801 was not done for patients prior to initiating allopurinol due to the limited availability and cost concerns for the patients. However, we followed the drug reactions of all our patients.

### Conclusion

In conclusion, based on our results, allopurinol and ranolazine are the useful, well-tolerated, and safe antianginal options for eligible symptomatic patients with a history of angioplasty. However, they showed different effects on some of studied outcomes. Therefore, the precise differences in their effects need to be further explored.

## Abbreviations

CARISA: Combination Assessment of Ranolazine in Stable Angina
ERICA: Efficacy of Ranolazine in Chronic Angina
MERLIN-TIMI: Metabolic Efficiency With Ranolazine for Less Ischemia in Non–ST-Segment Elevation Acute Coronary Syndromes
PCI: percutaneous coronary intervention
QOL: quality of life
TERISA: Type 2 Diabetes Evaluation of Ranolazine in Subjects with Chronic Stable Angina

## Data Availability

The data that support the findings of this study are available on request from SB. The data are not publicly available due to privacy/ethical restrictions.
